# Early Warning of Resistance to Bt Toxin Vip3Aa in *Helicoverpa zea*

**DOI:** 10.3390/toxins13090618

**Published:** 2021-09-02

**Authors:** Fei Yang, David L. Kerns, Nathan S. Little, José C. Santiago González, Bruce E. Tabashnik

**Affiliations:** 1 Department of Entomology, Texas A&M University, College Station, TX 77843, USA; dlkerns@tamu.edu (D.L.K.); josec.santiago@tamu.edu (J.C.S.G.); 2 USDA Agricultural Research Service, Stoneville, MS 38776, USA; nathan.little@usda.gov; 3 Department of Entomology, University of Arizona, Tucson, AZ 85721, USA

**Keywords:** *Bacillus thuringiensis*, resistance monitoring, Vip3Aa, sustainability, genetically engineered crop, corn, cotton

## Abstract

Evolution of resistance by pests can reduce the benefits of crops genetically engineered to produce insecticidal proteins from *Bacillus thuringiensis* (Bt). Because of the widespread resistance of *Helicoverpa zea* to crystalline (Cry) Bt toxins in the United States, the vegetative insecticidal protein Vip3Aa is the only Bt toxin produced by Bt corn and cotton that remains effective against some populations of this polyphagous lepidopteran pest. Here we evaluated *H. zea* resistance to Vip3Aa using diet bioassays to test 42,218 larvae from three lab strains and 71 strains derived from the field during 2016 to 2020 in Arkansas, Louisiana, Mississippi, Tennessee, and Texas. Relative to the least susceptible of the three lab strains tested (BZ), susceptibility to Vip3Aa of the field-derived strains decreased significantly from 2016 to 2020. Relative to another lab strain (TM), 7 of 16 strains derived from the field in 2019 were significantly resistant to Vip3Aa, with up to 13-fold resistance. Susceptibility to Vip3Aa was significantly lower for strains derived from Vip3Aa plants than non-Vip3Aa plants, providing direct evidence of resistance evolving in response to selection by Vip3Aa plants in the field. Together with previously reported data, the results here convey an early warning of field-evolved resistance to Vip3Aa in *H. zea* that supports calls for urgent action to preserve the efficacy of this toxin.

## 1. Introduction

Transgenic crops producing insecticidal proteins from *Bacillus thuringiensis* (Bt) can provide safe and effective control of some key pests [1,2,3,4]. In 2019, farmers planted such Bt crops on 109 million hectares in 27 countries [5]. However, the benefits of Bt crops are diminished when pests adapt to Bt toxins [6,7,8,9,10]. In this context, field-evolved resistance is defined as a genetically based decrease in susceptibility of an insect population to a Bt toxin caused by selection in the field [7]. Field-evolved resistance has two categories: practical resistance and early warning of resistance [6]. Practical resistance is field-evolved resistance that decreases the efficacy of the Bt crop in the field and has practical consequences for pest control [8]. An additional criterion for practical resistance is that more than 50% of individuals in a population are resistant [8]. Early warning of resistance includes all cases of field-evolved resistance that do not meet the additional criteria for practical resistance [6]. For both categories of field-evolved resistance, at least one field population of the pest must meet the relevant criteria.

Some populations of at least nine major lepidopteran and coleopteran pests have evolved practical resistance to Bt crystalline (Cry) toxins produced by transgenic crops [6,9,10], including *Helicoverpa zea* (corn earworm and bollworm), one of the most damaging crop pests in the United States [3,11]. Throughout much of the United States where this polyphagous lepidopteran is an important pest, it has evolved practical resistance to the Cry toxins produced by Bt corn and cotton, including Cry1Ab, Cry1Ac, Cry1A.105, Cry1Fa, and Cry2Ab [12,13,14,15,16,17,18,19,20,21,22]. Driven in part by this resistance, farmers have planted Bt corn and cotton that produce the Bt vegetative insecticidal protein Vip3Aa in addition to various Cry proteins [15,23].

**Figure 1:**
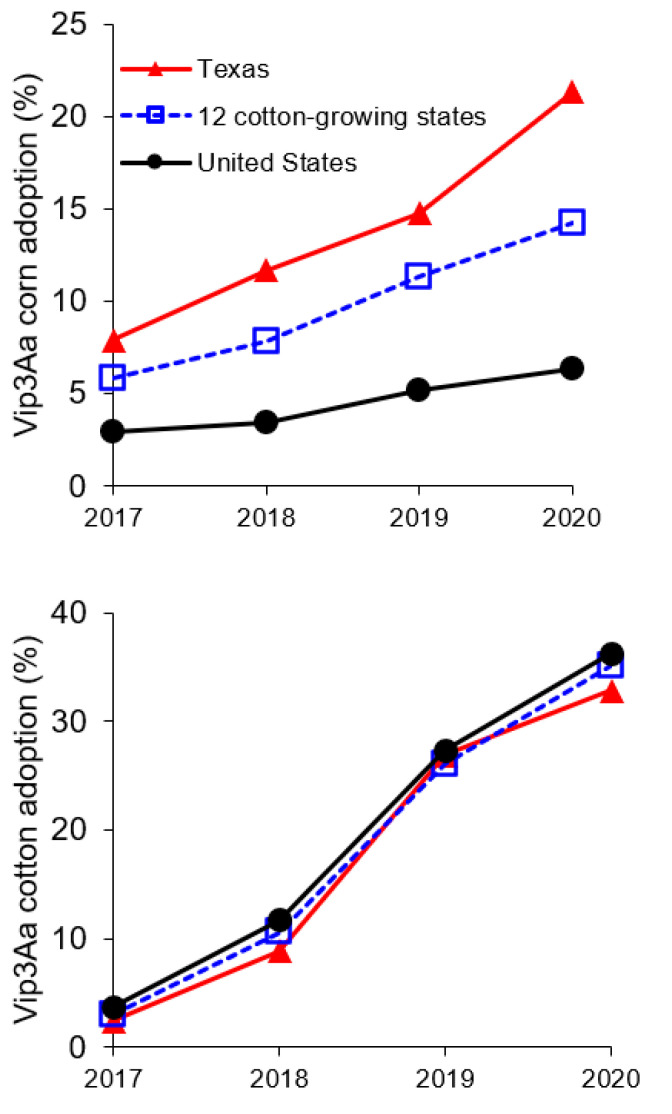
Percentage of corn or cotton hectares planted with corn or cotton producing Vip3Aa. ABSTC provided the data for Vip3Aa seed sales for 12 cotton-growing states (including Texas) and for the entire United States (see Supplementary Methods and Tables S1–S6 for details).

In the United States, Vip3Aa-producing corn and cotton were first registered in 2008 and 2009, respectively [24], but were not widely adopted until recently (Figure 1) [25,26]. Vip3 proteins are toxic to some lepidopteran larvae and have striking structural homology to Cry toxins, despite sharing no primary sequence similarity [27]. Like Cry toxins, Vip3 toxins are thought to kill insects by binding to specific receptors in the midgut [27,28]. Because Vip3 and Cry toxins do not bind to the same midgut receptors, strong cross-resistance between them is unlikely [29]. Indeed, analysis of 48 paired observations in 34 strains of eight species of lepidopteran pests revealed weak cross-resistance between Cry1 and Vip3 toxins and no cross-resistance between Cry2 and Vip3 toxins [30].

In two strains of *H. zea* selected in the lab for >50-fold resistance to Cry1Ac, the concentration of Vip3Aa killing 50% of larvae (LC_50_) did not increase in one strain [31]. In the other strain, however, the LC_50_ of Vip3Aa increased by 1.6-fold, revealing statistically significant but weak cross-resistance [32]. Because of the widespread practical resistance of *H. zea* to Cry toxins, Vip3Aa is the only Bt toxin in transgenic corn and cotton that remains effective against some populations of this pest in the United States [22].

Despite no strong cross-resistance between Cry toxins and Vip3Aa and the initial high efficacy of Vip3Aa against *H. zea* [33,34], some previously reported evidence suggests this pest has begun to evolve resistance to Vip3Aa in the southern United States [18,19,20,22,35]. For example, in a 2018 field trial at the Texas A&M University (TAMU) field laboratory in Snook, Texas, *H. zea* larvae damaged 67.5% of corn producing Cry1Ab + Cry1Fa + Vip3Aa [19]. Also, for two strains of *H. zea* derived in 2018 from Bt corn at the site of the field trial, the LC_50_ of Vip3Aa was 21 times higher for the strain from corn containing Vip3Aa than for the strain from corn without Vip3Aa [19].

Here, to evaluate the status of *H. zea* resistance to Vip3Aa, we analyzed data from diet bioassays of three lab strains and 71 strains derived from the field from 2016 to 2020 in Arkansas, Louisiana, Mississippi, Tennessee, and Texas (AR, LA, MS, TN, and TX). Together with previously reported data, the results here provide strong evidence of an early warning of field-evolved resistance to Vip3Aa in *H. zea*.

## 2. Results

We conducted 80 diet bioassays testing 42,218 larvae from three long-term lab strains (BZ, SIMRU, and TM) and 71 strains derived in 2016 to 2020 from field populations in AR, LA, MS, TN, and TX (Table 1, Table 2 and Table 3, Supplementary Figure S1). Bioassays were performed at TAMU, except in 2016 when all seven were done at Louisiana State University (LSU, Table 1) and in 2019 when BZ, TM, and 16 field-derived strains were tested at TAMU, while BZ and 10 field-derived strains were tested at the Southern Insect Management Research Unit (SIMRU), USDA-ARS in Stoneville, MS (Table 3). The units for the LC_50_ values reported below are micrograms (μg) Vip3Aa per cm^2^ diet (with 95% fiducial limits (FL)).

### 2.1. Variation in Susceptibility to Vip3Aa among Lab Strains of H. zea

Of the three lab strains tested, BZ was least susceptible to Vip3Aa. In two of the three direct comparisons between BZ and other lab strains tested at the same lab in the same year, BZ was significantly less susceptible than the other lab strains based on the conservative criterion of no overlap of the 95% FL of the LC_50_ values. In 2016, the LC_50_ of Vip3Aa was 2.8 times higher for BZ than for the SIMRU lab strain (Table 1); in 2019, it was 4.1 times higher for BZ than for the TM lab strain (Table 3). In the third direct comparison, the LC_50_ of Vip3Aa was 1.3 times higher for BZ than TM in 2018, which is not statistically significant (Table 2). In addition, the LC_50_ of Vip3Aa for BZ tested at SIMRU was 2.4 times higher than the LC_50_ of Vip3Aa for TM tested at TAMU (Table 3). No significant difference occurred in the LC_50_ of Vip3Aa for BZ between the tests in 2019 conducted at TAMU and the tests conducted at SIMRU.

We calculated the Vip3Aa resistance ratio (RR) as the LC_50_ of each strain divided by the LC_50_ of a lab strain tested in the same year in the same lab. We used the lab strain BZ as a standard for comparisons based on data throughout the study because it was the only lab strain tested in each year and at LSU, TAMU, and SIMRU. However, because BZ was the least susceptible of the three lab strains, comparisons with BZ tend to underestimate the magnitude and extent of resistance. Thus, to obtain results less likely to underestimate resistance, we also calculated RRs relative to the SIMRU lab strain in 2016 and relative to the TM lab strain for field-derived strains tested at TAMU in 2018 and 2019 (Table 1, Table 2 and Table 3). TM had nearly identical LC_50_ values in the two years it was tested: 0.16 (0.11, 0.25) in 2018 and 0.17 (0.14, 0.21) in 2019 (Table 2 and Table 3).

### 2.2. Resistance to Vip3Aa in Field-Derived Strains of H. zea Relative to Lab Strains

**Figure 2:**
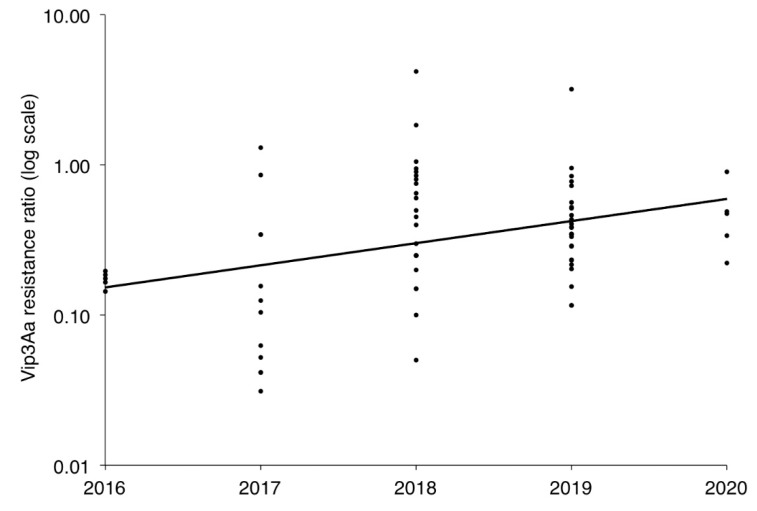
Increase from 2016 to 2020 in the Vip3Aa resistance ratio relative to the BZ lab strain for 71 field-derived strains of *Helicoverpa zea*. Linear regression: log(y) = 0.14X − 282; *R*^2^ = 0.12; df = 69; *p* = 0.003.

For the 71 field-derived strains, the Vip3Aa RR relative to BZ increased significantly from 2016 to 2020 (Figure 2). This genetically based decrease in susceptibility over time provides evidence of field-evolved resistance to Vip3Aa. Based on comparisons to BZ within each year, the LC_50_ values of Vip3Aa were significantly higher than BZ for 2 of the 71 field-derived strains: one established in 2018 from Snook, TX and the other in 2019 from Stoneville, MS (Table 2 and Table 3). The RRs relative to BZ for these strains were 4.2 and 3.2, respectively. Both strains were started with larvae collected from corn producing Cry1Ab + Cry1Fa + Vip3Aa.

For the 39 field-derived strains tested at TAMU in 2018 and 2019, the LC_50_ values were significantly higher than the TM lab strain for 1 of the 23 strains started in 2018 and 7 of the 16 strains started in 2019 (Table 2 and Table 3). The proportion of field-derived strains tested at TAMU with LC_50_ values significantly higher than TM increased 10-fold from 2018 (0.043) to 2019 (0.44) (Fisher’s exact test; *p* = 0.004). The maximum RRs relative to TM were 5.3 in 2018 and 13.0 in 2019, which were recorded for the two strains described above derived from corn producing Cry1Ab + Cry1Fa + Vip3Aa (Table 2 and Table 3).

We were able to establish five strains from *H. zea* collected in 2018 and 2019 on plants producing Vip3Aa, which reflects the substantial presence of the pest on these plants (mean: 118 pupae obtained per strain; range: 43 to 240; Table 2 and Table 3). In addition to the two strains described above, we derived strains in 2018 from Grant, LA from cotton producing Cry1Ac + Cry1Fa + Vip3a; and in 2019 from Snook, TX from cotton producing Cry1Ac + Cry2Ab + Vip3Aa and corn producing Cry1Ab + Cry1Fa + Vip3Aa (Table 2 and Table 3).

### 2.3. Resistance to Vip3Aa in H. zea from Vip3Aa Plants Relative to Non-Vip3Aa Plants

Providing direct evidence of evolution of resistance in response to selection imposed by exposure to Vip3Aa in the field, Vip3Aa RRs were higher for strains derived from Vip3Aa-producing plants than for comparable strains from plants that did not produce Vip3Aa. For the 39 field-derived strains tested at TAMU during 2018 and 2019, the Vip3Aa RRs relative to TM were 5.7-fold higher for the five strains from Vip3Aa plants (3.3) than for the 34 strains from non-Vip3Aa plants (0.6) (*t*-test of log-transformed RRs; *t* = 3.8; df = 37; *p* = 0.0005).

In addition, two of the five strains derived from Vip3Aa plants can be compared directly with strains derived from non-Vip3Aa plants from the same field site during the same season. Among the three strains established from Snook, TX in 2018, the LC_50_ for the strain from Cry1Ab + Cry1Fa + Vip3Aa corn was 21 times higher than for the strain from Cry1A.105 + Cry2Ab corn, as reported previously [19], and 2.3 times higher than for the strain from Cry1Ac + Cry1Fa cotton (Table 2). For the two strains established from Stonesville, MS in 2019, the LC_50_ for the strain from Cry1Ab + Cry1Fa + Vip3Aa corn was 28 times higher than for the strain from Cry1A.105 + Cry2Ab corn (Table 3).

In contrast with the results above for the 39 field-derived strains tested at TAMU during 2018 and 2019, no significant difference in Vip3Aa RRs relative to TM for these 39 strains occurred between the strains from Bt plants (*n* = 28) versus non-Bt plants (*n* = 11) (mean for Bt: 0.83 and non-Bt: 0.81; *t*-test of log-transformed RRs, *t* = 0.04, df = 37, *p* = 0.97). These results imply that selection for resistance to Vip3Aa was imposed specifically by Vip3Aa plants rather than by Bt plants generally, which is consistent with the lack of strong cross-resistance between Vip3Aa and Cry1 toxins or Cry2Ab [30].

## 3. Discussion

Several lines of evidence based on the diet bioassay results from this study support the conclusion that some *H. zea* field populations evaluated here have begun to evolve resistance to Vip3Aa. First, the Vip3Aa RR relative to lab strain BZ increased significantly from 2016 to 2020 in a collective analysis of the 71 strains derived from field populations throughout the five-state region studied here. Second, BZ was the least susceptible of the three lab strains tested and statistically significant resistance to Vip3Aa relative to BZ occurred in one strain derived from the field in 2018 and another in 2019. Third, relative to the lab strain TM, 7 of the 16 strains that were derived from field in 2019 and tested at TAMU were resistant to Vip3Aa, with a maximum RR of 13. Fourth, the proportion of field-derived strains tested at TAMU resistant to Vip3Aa relative to TM increased significantly from 0.043 in 2018 to 0.44 in 2019. Fifth, overall and in pairwise comparisons between strains established from the same field site and year, LC_50_ values of Vip3Aa were higher for strains derived from Vip3Aa plants than from non-Vip3Aa plants. This provides direct evidence of field-evolved resistance in response to selection by Vip3Aa in the field.

The response to selection noted above demonstrates substantial heritability of resistance to Vip3Aa in field populations. Such heritability is maximized at intermediate resistance allele frequencies and minimized at resistance allele frequencies close to either zero or one [37]. Based on an F_2_ screen of 114 families of *H. zea* derived from Snook, TX in 2019, the frequency of completely recessive resistance alleles that conferred over 500-fold resistance to Vip3Aa was estimated as 0.0065 (95% confidence interval = 0.0014–0.0157) [36,38]. Assuming Hardy–Weinberg equilibrium, the frequency of homozygous resistant individuals carrying such alleles in 2019 was 0.000042 (4.2 per 100,000). This frequency is expected to have essentially no effect on LC_50_ values in 2019. Moreover, because Vip3Aa plants accounted for considerably less than 100% of *H. zea* host plants in 2019 and 2020, we expect the frequency of these recessive resistance alleles was also too low to substantially affect LC_50_ values in 2020. For example, the expected frequency of individuals homozygous for these recessive resistance alleles in 2020 is 0.000060 (6.0 per 100,000) based on a population genetic model under the pessimistic assumptions that these alleles confer complete resistance of homozygotes to Vip3Aa plants, they carry no fitness cost on non-Vip3Aa plants, and refuges of non-Vip3Aa plants accounted for 10% of all *H. zea* host plants (see Methods). Thus, we hypothesize that the resistance observed in the diet bioassays here was not caused primarily by the recessive resistance alleles isolated from the F_2_ screen, but rather by more common alleles with smaller effects that are probably not recessive.

It is useful to compare the results from the diet overlay bioassays conducted here with results from the four previous studies that used similar methods to assess responses of *H. zea* larvae to Vip3Aa [21,22,39,40]. Niu et al. [22] used diet overlay bioassays to compare the LC_50_ of Vip3Aa they measured for the BZ strain (0.40 μg per cm^2^ diet, 95% FL = 0.30, 0.51) versus 29 strains derived from the field in 2018 and 2019 from seven southern states (Arkansas, Florida, Georgia, Louisiana, Mississippi, North Carolina, and South Carolina). Based on no overlap with the 95% FL of the LC_50_ of BZ, they reported the LC_50_ of Vip3Aa was significantly higher for 21% (6 of 29) of their field-derived strains. The maximum RR relative to BZ was 9.0, which was found for a strain derived from Cry1A.105 + Cry2Ab corn from Lonoke, AR in 2019. Their analysis tends to underestimate the extent and magnitude of resistance because non-overlap of 95% FL is a conservative statistical test and our results show that susceptibility to Vip3Aa was lower for the BZ strain than for the other lab strains. Moreover, five of their six field-derived strains with significant resistance to Vip3Aa relative to BZ were started with *H. zea* from non-Vip3Aa host plants. Of the 29 field-derived strains they evaluated, the only one derived from a Vip3Aa plant came from cotton producing Cry1Ac + Cry1Fa + Vip3Aa in Grant, LA and was significantly resistant relative to BZ (RR = 2.3).

In a related paper with the same senior author and bioassay methods as Niu et al. [22], Guo et al. [21] compared the LC_50_ value of Vip3Aa they recorded for BZ (1.14 μg per cm^2^ diet; 95% FL = 0.58, 1.69) versus 11 strains derived in 2018 and 2019 from the Louisiana State University Agricultural Center (LSUAC) near Winnsboro, LA. Relative to BZ, 8 of their 11 field-derived strains had significantly lower LC_50_ values, and none had a significantly higher LC_50_ value. Even relative to the lower LC_50_ of BZ reported by Niu et al. [22] noted above, only 1 of the 11 strains had a significantly higher LC_50_ value. These results showed that the population studied by Guo et al. [21] was not resistant to Vip3Aa relative to BZ. These results also suggest that the resistance relative to BZ in 6 of the 29 field-derived strains reported by Niu et al. [22] reflects genetic variation among populations rather than the random experimental error that occurs in repeated tests of the same population as reported by Guo et al. [21]. Because Guo et al. [21] found only one live *H. zea* larva on 1200 ears of corn producing Cry1Ab + Vip3Aa and the only other corn in the plots they sampled was non-Bt corn, we infer that all or nearly all of the larvae used to start their 11 field-derived strains were collected from non-Bt corn.

Leite et al. [39] used diet overlay bioassays to assess baseline variation in *H. zea* susceptibility to Vip3Aa in Brazil before 2014 when Vip3Aa plants were not widely grown there. The mean LC_50_ was 0.13 μg Vip3Aa per cm^2^ diet (range: 0.04–0.21) for the six field-derived strains they tested. Although they did not test a lab strain, all of the LC_50_ values they reported for the six field-derived strains are significantly lower than the LC_50_ of BZ reported by Niu et al. [22].

Before Bt crops producing Vip3Aa were registered in the United States, Ali and Luttrell [40] also used diet bioassays to evaluate *H. zea* susceptibility to Vip3Aa. Based on results from experiments using the diet overlay bioassay methods followed in our study and the three other studies summarized above [21,22,39], they reported values for their lab strain LabZA of 0.10 (0.08, 0.13) for LC_50_ and 0.08 (0.06, 0.10) for MIC_50_ (both in μg Vip3Aa per cm^2^ diet; see Methods for details). Both of these values for LabZA are significantly lower than the LC_50_ of BZ of 0.40 (0.30, 0.51) reported by Niu et al. [22]. Based on diet incorporation bioassays (which differ from diet overlay bioassays), they reported that none of the field-derived strains derived in 2006 and 2007 from the field in Alabama, Arkansas, Louisiana, North Carolina, and Texas had an LC_50_ value significantly greater than LabZA tested in the same year. Despite the lower susceptibility of BZ than LabZA, the proportion of field-derived strains with significant resistance to Vip3Aa was significantly higher in the 2018–2019 study by Niu et al. [22] using BZ as the standard (6 of 29) than in the 2006–2007 baseline study [40] using LabZA as the standard (0 of 27) (Fisher’s exact test; *p* = 0.02). These results support the hypothesis that some of the field populations studied by Niu et al. [22] evolved resistance to Vip3Aa.

Although the results from our study and from Niu et al. [22] meet the criteria for early warning of resistance, the category of practical resistance has the following additional criteria: resistance reduces the efficacy of the Bt crop and has practical consequences for pest management, and more than 50% of individuals in a population are resistant [8]. To evaluate the potential for reduced efficacy, we compared the number of larvae per ear in Vip3Aa corn relative to related non-Bt corn reported in four previous studies where efficacy can be clearly attributed to Vip3Aa (Supplementary Table S7 and Methods). This ratio provides an estimate of the frequency of phenotypic resistance [41]. This ratio was 0.85 in a 2018 field trial in Snook, TX, where the diet bioassay results in the same year provide evidence of field-evolved resistance to Vip3Aa [19]. The ratio of 0.85 is 14 to 1062 times higher than the ratios from the other three studies: 0.06 for 2006 and 0.04 for 2007 in the baseline study of Burkness et al. [33], 0.06 and 0.01 for two field sites in Louisiana in 2018 [17], and 0.0008 for Winnsboro, LA in 2018 and 2019 [21] (One-sample *t*-test of log-transformed data; *t* = 5.6; df = 4; *p* = 0.005). These ratios yield values of Vip3Aa efficacy of 15% for Snook in 2018 versus 94% to 99.9% for the other studies. Thus, in terms of decreasing the number of *H. zea* larvae per ear, the efficacy of Vip3Aa was reduced at Snook in 2018 relative to the other studies. However, the field data from Snook in 2018 also show 93% efficacy of Vip3Aa in terms of reducing the damaged area per ear [19], suggesting minimal practical consequences.

In addition, based on survival at a concentration of 3 μg Vip3Aa per cm^2^ diet as the criterion for resistance [36], none of the field-derived strains we tested met the criterion of having more than 50% resistant individuals. Niu et al. [22] reported that a strain derived in 2019 from Lonoke, AR had an LC_50_ of 3.60 μg Vip3Aa per cm^2^ diet, which suggests it met this criterion, but they proposed a diagnostic concentration of 5 to 10 μg Vip3Aa per cm^2^ diet. Leite et al. [39] suggested a diagnostic concentration of 6.4 μg Vip3Aa per cm^2^ diet based on their data from Brazil. We favor the lower concentration used by Yang et al. [36] as a diagnostic concentration or lower concentrations, because they are likely to detect resistance sooner than the higher concentrations. More importantly, switching from measurement of LC_50_ values to survival at a diagnostic concentration can be more efficient for detecting resistance when it first evolves in the field [42].

In summary, we conclude that our results and related work provide strong evidence of field-evolved resistance that clearly meets the criteria for early warning of resistance but not practical resistance. Rather than focusing on the somewhat arbitrary distinction between these two categories, we emphasize our agreement with other scientists that action is needed now to preserve the efficacy of Vip3Aa against *H. zea* in the United States [20,43,44,45]. One option is to prohibit selling and planting field corn hybrids that produce Vip3Aa in the cotton-growing regions of the United States [44,45]. This would limit selection for resistance to Vip3Aa in corn, where *H. zea* is not a major economic pest, and thereby help to preserve its efficacy against *H. zea* in cotton where it is a major economic pest [44,45,46]. This approach was unanimously recommended by the Scientific Advisory Panel convened by the US EPA in 2018 to address lepidopteran resistance to Bt crops [44]. It is also supported by the National Cotton States Arthropod Pest Management Working Group and by 36 public sector entomologists who participate in Multistate Research Project NC246: Ecology and Management of Arthropods in Corn [45]. The evidence reported here and related data highlight the urgency to implement these recommendations to sustain the efficacy of Vip3Aa against *H. zea.*

## 4. Materials and Methods

### 4.1. Strains of H. zea

We tested three lab strains (BZ, SIMRU, and TM) and 71 strains of *H. zea* derived from the field in AR, LA, MS, TN, and TX during 2016 to 2020. Strains were reared at LSU, TAMU, and SIMRU as described previously, with larvae reared on diet without Bt toxins or other insecticides [38]. We obtained the BZ strain (called CBW-BZ-SS in [19]) from Benzon Research Inc., Carlisle, PA. The TM strain (called SS in [36]) was started from more than 150 larvae collected from non-Bt corn in May 2016 at the LSUAC near Winnsboro, LA. The SIMRU strain has been maintained in the lab since 1971 [18].

To start field-derived strains, third to fifth instar larvae of *H. zea* were collected from Bt and non-Bt host plants in the field (Table 1, Table 2 and Table 3). The Bt host plants include Bt corn: Intrasect, Obsession sweet corn, Leptra, SmartStax (STX), VT DoublePRO (VT2P), and VT TriplePro (VT3P); and Bt cotton: Bollgard II (BG2), Bollgard 3 (BG3), TwinLink, Widestrike (WS), and Widestrike3 (WS3). The non-Bt host plants include corn, cotton, crimson clover (*Trifolium incarnatum*), grain sorghum (*Sorghum bicolor*), and soybean (*Glycine max*). The field-collected larvae were put in 30 mL plastic cups (Solo^®^ Mason, MI) containing Stonefly Heliothis diet (Ward’s Natural Science, Rochester, NY). The cups were put in insulated boxes containing frozen blue ice and other packing materials, then sent via overnight delivery to LSU, TAMU or SIMRU where larvae were transferred one per cup into 30 mL cups containing fresh diet. To synchronize development of the field-collected larvae, they were sorted by instar and reared at different temperatures to accelerate the growth of earlier instars relative to later instars until all larvae were at the same developmental stage. We put pupae in 20 L mesh cages (Seville Classics Inc., Torrance, CA, USA) containing ~200 g vermiculite (Sun Gro, Pine Bluff, AR, USA) and a 10% honey–water solution to feed moths that emerged. Cages were maintained at 26 ± 1 °C, ∼60% relative humidity, and 16 h light: 8 h dark.

### 4.2. ELISA Tests for Vip3Aa in Putative Vip3Aa Plants

To check for Vip3Aa in putative Vip3Aa corn and cotton plants from which insects were collected at five field sites in 2018 and 2019 (Table 2 and Table 3), we used ELISA tests according to the manufacturer’s instructions (EnviroLogix, Quantiplate^TM^ kits, Portland, ME). From each of the five sites, we tested three to five plants expected to produce Vip3Aa and a similar number that were not expected to produce Vip3Aa. All plants tested that were expected to produce Vip3Aa yielded positive results in the ELISA tests, confirming the presence of Vip3Aa. No Vip3Aa was detected in the plants that were not expected to produce Vip3Aa.

### 4.3. Diet Overlay Bioassays

We conducted 80 diet overlay bioassays to determine the susceptibility to Vip3Aa of 71 field-derived strains and three lab strains (total of 9 bioassays: BZ tested six times, TM tested in 2018 and 2019, and SIMRU tested in 2016). The 2016 bioassays were performed at the LSU Department of Entomology. The other bioassays were performed at TAMU, except for 11 conducted at SIMRU in 2019 for BZ and 10 field-derived strains (Table 3). For 68 of the 71 field-derived strains, we tested the first-generation (F_1_) progeny of the field-collected insects. Because of insufficient numbers of F_1_, we tested the F_2_ progeny for three strains from Bt cotton (from Cry1Ac + Cry2Ab cotton from Alexandria, LA in 2016 and Wharton, TX in 2018; and from Cry1Ab + Cry2Ae cotton from Stoneville, MS in 2019 and tested at SIMRU).

To test each strain, we used concentrations of 0, 0.01, 0.0316, 0.1, 0.316, 1.0, and 3.16 μg Vip3Aa per cm^2^ diet. Each combination of insect strain by Vip3Aa concentration was replicated four times with 16 to 32 larvae per replicate. We put 0.8 mL of liquid *H. zea* diet (Southland Products Inc., Lake Village, AR, USA) per well in 128-well bioassay trays (C-D International, Pitman, NJ, USA). After the diet cooled and solidified, 40 μL of a suspension containing an appropriate concentration of Vip3Aa in 0.1% Triton-X100 was overlaid on the diet surface of each well and allowed to dry. We put one neonate (<24 h old) on the diet surface of each well and covered all wells with vented lids (C-D International, Pitman, NJ, USA). We put the bioassay trays in an environmental chamber at 26 ± 1 °C, 50% relative humidity, and 14 h light: 10 h dark. After 7 days, we recorded the number of dead larvae and the instar of live larvae.

### 4.4. Vip3Aa

We used Vip3Aa19 in 2016 and 2017, Vip3Aa51 in 2018, and Vip3Aa39 in 2019 and 2020. Vip3Aa19 was provided by Syngenta and Vip3Aa51 by BASF (both at Research Triangle Park, NC, USA). Juan Luis Jurat-Fuentes, University of Tennessee, provided Vip3Aa39. The amino acid sequences of Vip3Aa39 and Vip3Aa51 are identical and 97.1% similar to Vip3Aa19 [47], which is produced by Bt cotton. Relative to Vip3Aa20 produced by Bt corn, the amino acid sequence similarity is 99.9% for Vip3Aa19 and 97.1% for Vip3Aa39 and Vip3Aa51 [47]. Because the amino acid sequence similarity among these toxins is 97.1% to 100%, we refer to all of them as Vip3Aa. However, rather than assuming these toxins had identical potency against *H. zea,* we recognize that potency could have varied among them or even across years or between labs for the same toxin. Thus, we emphasize comparisons between strains that were tested with the same toxin in the same year and the same lab. See Results section for details of how we calculated RRs and made comparisons between strains.

### 4.5. Data Analysis

#### 4.5.1. Diet Bioassay Data

We used probit analysis [48] to calculate the concentration of Vip3Aa causing 50% larval mortality (LC_50_) and the corresponding 95% fiducial limits (FL) for each strain. Larval mortality was based on the number of dead larvae and live first instars and was adjusted for control mortality before the data were entered for probit analysis [38]. As done here, four previous studies of *H. zea* responses to Vip3Aa use the term LC_50_ to refer to the metric based on the number of dead larvae and live first instars [21,22,31,39]. Welch et al. [32] call this EC_50_. Ali and Luttrell [40] call it MIC_50_ and use the term LC_50_ for larval mortality only (not including live first instars). Three of the 80 values of the LC_50_ of Vip3Aa reported in Table 1, Table 2 and Table 3 were reported previously: two for strains derived in Snook, TX in 2018; one from corn producing Cry1Ab + Cry1Fa + Vip3Aa; one from corn producing Cry1A.105 + Cry2Ab (called CEW-TX-Leptra-2018 and CEW-TX-VGT3P-2018, respectively, in [19]; and one for the TM lab strain tested in 2019 (called SS in [36]).

We considered two values of LC_50_ significantly different if their 95% FL did not overlap, which is a conservative criterion [49,50]. In addition to such pairwise comparisons, we conducted a regression analysis and *t*-tests using log-transformed values of RRs.

#### 4.5.2. Efficacy of Vip3Aa Corn

We calculated the number of larvae per ear in Bt corn relative to comparable non-Bt corn using data from four previous field studies [17,19,21,33] from which we can reasonably infer that Vip3Aa was the sole or primary Bt toxin reducing the number of *H. zea* larvae per ear (Supplementary Table S7). From Burkness et al. [33], the data we used for Bt corn are for hybrids producing only Vip3Aa. In Yang et al. [19] and Kaur et al. [17], Cry1Ab + Cry1Fa corn did not have fewer larvae per ear than related non-Bt corn, so the reported efficacy of Cry1Ab + Cry1Fa + Vip3Aa corn can be attributed to Vip3Aa. Guo et al. [21] reported high levels of resistance to Cry1Ab, so the efficacy they reported for Cry1Ab + Vip3Aa corn can be attributed primarily or entirely to Vip3Aa.

We used a one-sample *t*-test of the log-transformed data to assess the difference between the results from Snook, TX in 2018 and the other previous results in the ratio of larvae per ear in Bt corn relative to non-Bt corn. We quantified the efficacy of Bt corn in terms of reducing the number of larvae per ear as 100% multiplied by (1 − [LBt/LNon-Bt], where LBt and LNon-Bt are the number of larvae per ear for Bt and comparable non-Bt corn, respectively [8]. Thus, efficacy is 0% when LBt equals LNon-Bt, and 100% when LBt equals 0.

Dively et al. [20] reported mean ratios from 19 to 27 trials per time period for the number of larvae per ear in sweet corn producing Cry1Ab + Vip3Aa relative to its isoline non-Bt corn, which were 0.006 in 2007–2014, 0.008 in 2017, 0.024 in 2018, and 0.014 in 2019. Because these ratios may be affected by variation in efficacy of Cry1Ab across years and locations, we did not include them in the analysis reported in the Discussion. However, additional analyses that include either all of the data from Dively et al. [20] or just their data from 2017 to 2019 when we expect the efficacy of Cry1Ab was limited, confirm the conclusion reported in the Discussion that the ratio of larvae per ear in Vip3Aa corn relative to non-Bt corn for Snook, TX in 2018 was significantly higher than the previously reported ratios (One-sample *t*-tests of log-transformed data; *p* < 0.0001 for both additional analyses).

### 4.6. Population Genetic Modeling

We used a previously described deterministic population genetic model of *H. zea* [51]. We assumed resistance was controlled by a single locus with a recessive allele for resistance [36,38] and another allele for susceptibility. The initial frequency of the resistance allele was set at 0.0065, as estimated for 2019 for *H. zea* in Texas [38]. The dominance parameter *h* was set to zero, indicating completely recessive resistance, as empirically determined [36]. We assumed the fitness of resistant homozygotes was 1.0 for individuals that developed as larvae on plants with or without Vip3Aa. This represents complete resistance to Vip3Aa and no fitness cost associated with resistance on non-Vip3Aa plants. Non-Vip3Aa plant refuges accounted for 10% of all host plants. To predict the resistance allele frequency in 2020, we used the model to simulate one year. The assumptions we evaluated are pessimistic, because the expected resistance allele frequency would be lower in 2020 if the resistant homozygotes were not completely resistant to Vip3Aa plants, the resistance carried a fitness cost, or non-Vip3Aa plants provided a refuge accounting for more than 10% of all host plants.

## Figures and Tables

**Table 1:** Responses to Vip3Aa of *H. zea* larvae from two lab strains (BZ and SIMRU) and 17 strains derived from the field in 2016 and 2017.

Host Plant ^a^	Bt Toxins in Host Plant ^a^	Field Site or Lab Strain Name	Pupae ^b^	Larvae ^c^	Slope ± SE	LC_50_ (95% FL) ^d^	RR vs. BZ ^e^	RR vs. SIMRU ^f^
**2016: LSU**								
Lab diet	None	BZ	/	957	2.8 ± 0.4	0.97 (0.85, 1.11)	1.0	2.8
Lab diet	None	SIMRU	/	958	1.9 ± 0.3	0.35 (0.22, 0.56)	0.4	1.0
Non-Bt corn	None	Toad Suck, AR	114	956	1.6 ± 0.2	0.17 (0.13, 0.23)	0.2	0.5
BG2 cotton	Cry1Ac + Cry2Ab	Alexandria, LA	37	945	1.8 ± 0.2	0.19 (0.15, 0.24)	0.2	0.5
VT2P corn	Cry1A.105 + Cry2Ab	Leland, MS	182	962	2.2 ± 0.1	0.14 (0.12, 0.16)	0.1	0.4
Grain sorghum	None	Jackson, TN	118	956	2.1 ± 0.2	0.16 (0.12, 0.21)	0.2	0.5
BG2 cotton	Cry1Ac + Cry2Ab	Jackson, TN	92	943	1.8 ± 0.2	0.18 (0.13, 0.23)	0.2	0.5
**2017: TAMU**								
Lab diet	None	BZ	/	895	2.8 ± 0.3	0.96 (0.86, 1.12)	1.0	/
Grain sorghum	None	Rohwer, AR	157	448	5.2 ± 1.2	1.25 (1.00, 1.57)	1.3	/
Non-Bt cotton	None	Alexandria, LA	131	448	2.4 ± 0.2	0.10 (0.08, 0.12)	0.1	/
TwinLink cotton	Cry1Ab + Cry2Ae	Alexandria, LA	107	448	1.7 ± 0.3	0.33 (0.19, 0.61)	0.3	/
BG2 cotton	Cry1Ac + Cry2Ab	Jonesville, LA	121	448	2.0 ± 0.2	0.15 (0.12, 0.18)	0.2	/
Non-Bt corn	None	Winnsboro, LA	186	895	2.1 ± 0.4	0.33 (0.18, 0.61)	0.3	/
BG2 cotton	Cry1Ac + Cry2Ab	Benoit, MS	69	448	1.3 ± 0.2	0.04 (0.03, 0.06)	0.04	/
BG2 cotton	Cry1Ac + Cry2Ab	Silver City, MS	75	448	3.2 ± 0.3	0.05 (0.04, 0.06)	0.1	/
VT2P corn	Cry1A.105 + Cry2Ab	Stoneville, MS	111	894	1.8 ± 0.2	0.06 (0.04, 0.07)	0.1	/
VT2P & non-Bt corn	Cry1A.105 + Cry2Ab	Starkville, MS	97	448	2.6 ± 0.3	0.04 (0.03, 0.05)	0.0	/
Obsession corn	Cry1A.105 + Cry2Ab	Milan, TN	135	448	2.3 ± 0.2	0.12 (0.10, 0.14)	0.1	/
WS cotton	Cry1Ab + Cry1Fa	Snook, TX	77	896	2.1 ± 0.4	0.03 (0.02, 0.04)	0.03	/
TwinLink cotton	Cry1Ab + Cry2Ae	Wharton, TX	20	895	5.7 ± 0.9	0.82 (0.73, 0.90)	0.9	/

^a^ Lab diet for the lab strains BZ and SIMRU; ^b^ Number of pupae obtained from field-collected larvae; ^c^ Number of larvae tested in bioassays; ^d^ Concentration killing 50% of larvae and its 95% fiducial limits (both in μg Vip3Aa per cm^2^ diet); ^e^ LC_50_ of strain divided by LC_50_ for BZ tested in the same year; ^f^ LC_50_ of strain divided by LC_50_ for SIMRU, calculated only for strains tested in 2016.

**Table 2:** Responses to Vip3Aa of *H. zea* larvae from two lab strains (BZ and TM) and 23 strains derived from the field in 2018 (all tested at TAMU).

Host Plant ^a^	Bt Toxins in Host Plant ^a^	Field Site or Lab Strain Name	Pupae ^b^	Larvae ^c^	Slope ± SE	LC_50_ (95% FL) ^d^	RR vs. BZ ^e^	RR vs. TM ^f^
Lab diet	None	BZ	/	448	1.5 ± 0.1	0.20 (0.16, 0.26)	1.0	1.3
Lab diet	None	TM	/	448	2.2 ± 0.4	0.16 (0.11, 0.25)	0.8	1.0
Intrasect corn	Cry1Ab + Cry1F	Little Rock, AR	130	448	2.1 ± 0.2	0.05 (0.04, 0.06)	0.3	0.3
and VT2P corn	Cry1A.105 + Cry2Ab2							
Non-Bt corn	None	Pine Bluff, AR	280	448	3.1 ± 0.3	0.13 (0.11, 0.16)	0.7	0.8
BG2 cotton	Cry1Ac + Cry2Ab	Alexandria, LA	300	448	2.1 ± 0.2	0.05 (0.04, 0.06)	0.3	0.3
WS3 cotton	Cry1Ac + Cry1Fa + Vip3Aa	Grant, LA	240	448	2.7 ± 0.4	0.12 (0.10, 0.16)	0.6	0.8
Crimson clover	None	Winnsboro, LA	300	448	2.5 ± 0.2	0.06 (0.05, 0.07)	0.3	0.4
Non-Bt corn	None	Winnsboro, LA	300	448	3.1 ± 0.3	0.05 (0.04, 0.06)	0.3	0.3
Soybean	None	Indianola, MS	120	448	2.5 ± 0.4	0.18 (0.12, 0.25)	0.9	1.1
Crimson clover	None	Natchez, MS	220	448	2.0 ± 0.3	0.19 (0.12, 0.31)	1.0	1.2
Obsession corn	Cry1A.105 + Cry2Ab	Jackson, TN	180	448	2.2 ± 0.3	0.21 (0.15, 0.31)	1.1	1.3
BG2 cotton	Cry1Ac + Cry2Ab	Jackson, TN	150	448	1.9 ± 0.3	0.01 (0.01, 0.02)	0.1	0.1
Non-Bt corn	None	Amarillo, TX	193	448	2.2 ± 0.4	0.15 (0.13, 0.18)	0.8	0.9
VT3P corn	Cry1A.105 + Cry2Ab	Snook, TX ^g^	300	448	2.8 ± 0.3	0.04 (0.03, 0.05)	0.2	0.3
WS cotton	Cry1Ac + Cry1Fa	Snook, TX	200	448	3.3 ± 1.0	0.37 (0.20, 0.71)	1.9	2.3
Leptra corn	Cry1Ab + Cry1Fa + Vip3Aa	Snook, TX ^h^	100	448	4.9 ± 1.0	0.84 (0.69, 0.97)	4.2 *	5.3 *
BG2 cotton	Cry1Ac + Cry2Ab	EI Campo, TX	28	448	2.8 ± 0.3	0.05 (0.04, 0.06)	0.3	0.3
Non-Bt corn	None	Los Indios, TX	150	448	2.5 ± 0.2	0.10 (0.08, 0.12)	0.5	0.6
Non-Bt corn	None	Lubbock, TX	272	448	4.2 ± 0.5	0.17 (0.15, 0.20)	0.9	1.1
VT2P corn	Cry1A.105 + Cry2Ab	Muleshoe, TX	210	448	1.8 ± 0.2	0.03 (0.02, 0.04)	0.2	0.2
Grain sorghum	None	Port Lavaca, TX	138	448	2.8 ± 0.3	0.09 (0.07, 0.11)	0.5	0.6
STX corn	Cry1A.105 + Cry1Fa + Cry2Ab	Thrall, TX	87	448	2.1 ± 0.2	0.08 (0.07, 0.10)	0.4	0.5
BG2 cotton	Cry1Ac + Cry2Ab	Wellington, TX	108	448	2.5 ± 0.3	0.03 (0.03, 0.04)	0.2	0.2
BG2 cotton	Cry1Ac + Cry2Ab	Wharton, TX	70	448	2.9 ± 0.6	0.02 (0.01, 0.03)	0.1	0.1
VT3P corn	Cry1A.105 + Cry2Ab	Wall, TX	103	448	2.3 ± 0.3	0.16 (0.12, 0.22)	0.8	1.0

^a^ Lab diet for the lab strains BZ and TM; ^b^ Number of pupae obtained from field-collected larvae; ^c^ Number of larvae tested in bioassays; ^d^ Concentration killing 50% of larvae and its 95% fiducial limits (both in μg Vip3Aa per cm^2^ diet); ^e^ LC_50_ of strain divided by LC_50_ for BZ; ^f^ LC_50_ of strain divided by LC_50_ for TM; ^g^ Data reported in [19], strain referred to there as CEW-TX-VT3P-2018; ^h^ Data reported in [19], strain referred to there as CEW-TX-Leptra-2018; * LC_50_ significantly greater for this strain than lab strains BZ and TM.

**Table 3:** Responses to Vip3Aa of *H. zea* larvae from two lab strains (BZ and TM) and 31 strains derived from the field in 2019 and 2020.

Host Plant ^a^	Bt Toxins in Host Plant ^a^	Field Site or Lab Strain Name	Pupae ^b^	Larvae ^c^	Slope ± SE	LC_50_ (95% FL) ^d^	RR vs. BZ ^e^	RR vs. TM ^f^
**2019: TAMU**								
Lab diet	None	BZ	/	448	3.7 ± 0.5	0.69 (0.56, 0.87)	1.0	4.1 *
Lab diet	None	TM ^g^	/	448	2.8 ± 0.3	0.17 (0.14, 0.21)	0.2	1.0
VT2P corn	Cry1A.105 + Cry2Ab	Lafayette Co., AR	120	448	2.8 ± 0.3	0.39 (0.33, 0.47)	0.6	2.3 *
VT2P corn	Cry1A.105 + Cry2Ab	Tillar, AR	115	448	2.5 ± 0.3	0.15 (0.13, 0.19)	0.2	0.9
VT2P corn	Cry1A.105 + Cry2Ab	Alexandria, LA	278	448	2.2 ± 0.2	0.23 (0.18, 0.28)	0.3	1.4
BG2 cotton	Cry1Ac + Cry2Ab	Alexandria, LA	180	448	2.5 ± 0.2	0.24 (0.20, 0.29)	0.3	1.4
VT2P corn	Cry1A.105 + Cry2Ab	Winnsboro, LA	198	448	2.5 ± 0.2	0.14 (0.12, 0.17)	0.2	0.8
Leptra corn	Cry1Ab + Cry1Fa + Vip3Aa	Stoneville, MS	82	448	1.6 ± 0.3	2.21 (1.27, 4.44)	3.2 *	13.0 *
VT2P corn	Cry1A.105 + Cry2Ab	Stoneville, MS	105	448	2.9 ± 0.3	0.08 (0.07, 0.10)	0.1	0.5
VT2P corn	Cry1A.105 + Cry2Ab	Starkville, MS	285	448	2.8 ± 0.3	0.16 (0.13, 0.19)	0.2	0.9
VT2P corn	Cry1A.105 + Cry2Ab	Jackson, TN	210	448	2.5 ± 0.3	0.32 (0.24, 0.44)	0.5	1.9 *
VT2P corn	Cry1A.105 + Cry2Ab	Hillsboro, TX	90	448	3.6 ± 0.4	0.30 (0.25, 0.35)	0.4	1.8 *
BG2 cotton	Cry1Ac + Cry2Ab	Jackson, TX	100	448	3.0 ± 1.3	0.20 (0.03, 0.17)	0.3	1.2
Non-Bt corn	None	Lubbock, TX	172	448	2.9 ± 0.3	0.28 (0.24, 0.34)	0.4	1.6 *
BG2 cotton	Cry1Ac + Cry2Ab	Navasota, TX	117	448	3.1 ± 0.3	0.08 (0.07, 0.10)	0.1	0.5
Leptra corn	Cry1Ab + Cry1Fa + Vip3Aa	Snook, TX	46	448	1.8 ± 0.2	0.66 (0.49, 0.89)	1.0	3.9 *
BG3 cotton	Cry1Ac + Cry2Ab + Vip3Aa	Snook, TX	123	448	1.8 ± 0.2	0.50 (0.40, 0.63)	0.7	2.9 *
Non-Bt corn	None	Wharton, TX	102	448	2.1 ± 0.2	0.24 (0.19, 0.29)	0.3	1.4
**2019: SIMRU**								
Lab diet	None	BZ	/	512	1.7 ± 0.3	0.41 (0.26, 0.68)	1.0	/
Non-Bt corn	None	Pickens, AR	246	512	2.3 ± 0.3	0.35 (0.26, 0.47)	0.8	/
Bt corn	Cry1A.105 + Cry2Ab	Leland, MS	238	512	1.7 ± 0.2	0.22 (0.16, 0.29)	0.5	/
Crimson clover	None	Grenada, MS	242	512	2.6 ± 0.3	0.10 (0.08, 0.12)	0.2	/
Crimson clover	None	Marks, MS	177	512	1.5 ± 0.2	0.21 (0.13, 0.35)	0.5	/
Non-Bt corn	None	Mound Bayou, MS	451	512	2.4 ± 0.3	0.14 0.11, 0.19)	0.3	/
Crimson clover	None	Olive Branch, MS	400	384	2.4 ± 0.4	0.12 (0.08, 0.17)	0.3	/
VT2P corn	Cry1A.105 + Cry2Ab	Rolling Fork, MS	240	512	2.1 ± 0.2	0.16 (0.12, 0.21)	0.4	/
Non-Bt corn	None	Stoneville, MS	282	512	2.4 ± 0.3	0.32 (0.24, 0.44)	0.8	/
TwinLink cotton	Cry1Ab + Cry2Ae	Stoneville, MS	79	512	1.9 ± 0.2	0.06 (0.04, 0.10)	0.2	/
Crimson clover	None	Warren County, MS	201	512	1.6 ± 0.2	0.16 (0.11, 0.22)	0.4	/
**2020: TAMU**								
Lab diet	None	BZ	/	448	3.2 ± 0.4	0.11 (0.09, 0.13)	1.0	/
VT2P corn	Cry1A.105 + Cry2Ab	Stoneville, MS	186	448	3.9 ± 0.5	0.06 (0.05, 0.06)	0.5	/
VT2P corn	Cry1A.105 + Cry2Ab	Pine Bluff, AR	135	448	1.3 ± 0.2	0.05 (0.03, 0.08)	0.5	/
VT2P corn	Cry1A.105 + Cry2Ab	Alexandria, LA	93	448	2.6 ± 0.5	0.03 (0.02, 0.03)	0.2	/
Non-Bt corn	None	Winnsboro, LA	210	448	3.7 ± 0.6	0.04 (0.03, 0.04)	0.3	/
VT2P corn	Cry1A.105 + Cry2Ab	Jackson, TN	178	448	3.4 ± 0.4	0.10 (0.09, 0.12)	0.9	/

^a^ Lab diet for the lab strains BZ and TM; ^b^ Number of pupae obtained from field-collected larvae; ^c^ Number of larvae tested in bioassays; ^d^ Concentration killing 50% of larvae and its 95% fiducial limits (both in μg Vip3Aa per cm^2^ diet); ^e^ LC_50_ of strain divided by LC_50_ for BZ tested in the same year; ^f^ LC_50_ of strain divided by LC_50_ for TM, calculated only for strains tested in 2019; ^g^ Data reported in Yang et al. [36], strain referred to there as SS; * LC_50_ significantly greater for this strain than lab strain BZ or TM, respectively, tested at TAMU in 2019.

## Data Availability

The data are available in the article and in the Supplementary Materials.

## Supplementary Materials

The following are available online at https://www.mdpi.com/article/10.3390/toxins13090618/s1, Supplementary Methods. Adoption of Vip3Aa corn and cotton. Tables S1–S6. Adoption of Vip3Aa corn and cotton. Table S7. Larvae per ear in Vip3Aa corn relative to comparable non-Bt corn in four previous field studies. Figure S1. Map of field sites for monitoring resistance of *H. zea* to Vip3Aa. References [25,26,52,53,54,55,56,57] are cited in the supplementary materials.
