# Supplementary material for: Early Warning of Resistance to Bt Toxin Vip3Aa in Helicoverpa zea
Source: Toxins (Basel). 2021 Sep 2;13(9):618. doi: 10.3390/toxins13090618 (PMC8473270; doi:10.3390/toxins13090618)
Supplement: Supplementary file 1 [file toxins-13-00618-s001.zip › toxins-1291424-SI.pdf]

Supplementary Materials: Early Warning of Resistance to Bt Toxin Vip3Aa in *Helicoverpa zea*

Fei Yang, David L Kerns, Nathan S. Little, José C. S. González and Bruce E. Tabashnik

Supplementary Methods: Adoption of Vip3Aa Corn and Cotton.

We calculated the percentage of acres of corn and cotton planted to Vip3Aa corn and cotton from 2017 to 2020 using the data ABSTC provided for bags of Vip3Aa seeds sold for 12 cotton-growing states (AL, AZ, AR, FL, GA, LA, MS, NM, NC, SC, TN, TX) and for the entire United States (Tables S1-S2). To convert bags of seeds sold to acres planted, we used the following conversion factors for acres per seed bag: 2.9 for corn for the 12 cotton states, 2.6 for corn for the entire U.S., and 5 for all cotton. For corn, the conversion factors are based on 80,000 seeds per bag, with means of 27,273 and 31,358 seeds per acre in the 12 cotton states and the entire U.S., respectively [52,53]. The conversion factor for cotton is based on 250,000 seeds per bag and 50,000 seeds per acre [54,55]. To calculate the percentage of corn and cotton planted with Vip3Aa corn and cotton, we divided the Vip3Aa acres by the total acres for each crop [56,57; Tables S3-S6).

For the calculations described above, we used the sales data provided by ABSTC without adjustment, except for three outlier values for corn in 2019. The 2019 outlier values were 28,992 for AZ; 7,766 for AR; and 20,548 for LA (Table S1). We adjusted these three values because relative to each state's mean for 2018 and 2020, the value for 2019 was 10.0-fold greater for AZ, 3.2-fold greater for LA, and 3.5-fold lower for AR. Thus, to calculate acres of Vip3Aa planted we used the means for 2018 and 2020: 2,898 for AZ, 27,441 for AR, and 6,384 for LA. This adjustment had a minor effect on the percentage adoption of Vip3Aa corn for the 12 cotton-growing states and the entire U.S.

For corn, Yu et al. [58] reported substantially higher rates of Vip3Aa adoption for 2015-2016 than those calculated here for 2017 (Fig. 1 and Table S5). In pairwise comparisons for the eight states included in both studies (AL, AR, GA, LA, MS, SC, TN, and TX) the percentages reported by Yu et al. [58] for 2015-2016 were a mean of 5.5 times higher than those calculated here for 2017. Because it is unlikely Vip3Aa adoption dropped from 2015-2016 to 2017, this discrepancy implies adoption was overestimated in the previous study, underestimated here, or both.

For cotton, Vip3Aa adoption percentages for the U.S. for 2017 to 2020 corresponded well between data reported by the USDA Agricultural Marketing Service (AMS) [59] and those calculated here. The percentages from the USDA AMS versus here were, respectively: 2017: 3.7 vs. 3.7, 2018: 14.1 vs. 11.7, 2019: 28.3 vs. 27.4, and 2019: 48.9 vs. 36.3 ($r^2 = 0.96$, df = 2, $P = 0.02$). On average for the four years, the percentages from USDA AMS were 1.1 times higher than those calculated here (range: 1.0 for 2017 to 1.3 for 2020).

Table S1. Vip3Aa corn seed bags sold. Source: ABSTC.

State or Region	2017	2018	2019	2020
AL	619	757	7,193	9912
AZ	1057	1525	28,992	4271
AR	18,742	26,590	7766	28,291
FL	5996	4761	5635	4145
GA	7668	6462	10,036	8725
LA	2793	7147	20,548	5621
MS	2523	7238	6347	8600
NM	1961	2816	3111	4535
NC	21,392	20,502	66,053	54,154
SC	5663	6311	19,324	26,342
TN	3478	6545	25,824	33,997
TX	66,989	88,766	127,470	165,581
12 cotton states	138,881	179,421	328,299	354,174
USA	1,026,107	1,173,270	1,786,801	2,221,836

Table S2. Vip3Aa cotton seed bags sold. Source: ABSTC.

State or Region	2017	2018	2019	2020
AL	3874	11,477	24,544	27,203
AZ	3002	6491	10,333	15,211
AR	1925	11,839	17,840	31,253
FL	213	723	2858	3763
GA	3588	29,480	64,559	96,750
LA	1519	5191	16,068	11,597
MS	3146	11,514	27,566	32,320
NM	10,369	2949	4016	3242
NC	1141	27,932	54,439	55,338
SC	2199	5355	12,998	15,088
TN	4502	12,759	20,813	18,977
TX	33,580	137,854	379,571	447,356
12 cotton states	69,058	263,564	635,605	758,098
USA	92,023	324,673	739,029	862,150

Table S3. Corn planted (1000 acres). Source: USDA NASS.

State or Region	2017	2018	2019	2020
AL	250	255	320	330
AZ	65	80	90	75
AR	620	660	770	620
FL	75	95	90	100
GA	290	325	395	420
LA	500	460	570	500
MS	520	480	660	510
NM	125	135	145	125
NC	890	910	990	1000
SC	350	340	380	400
TN	750	720	970	870
TX	2450	2200	2500	2250
12 cotton states	6885	6660	7880	7200
USA	90,167	88,871	89,700	90,819

Table S4. Cotton planted (1,000 acres). Source: USDA NASS.

State or Region	2017	2018	2019	2020
AL	435	510	540	450
AZ	160	160	160	125
AR	445	485	620	525
FL	99	117	112	98
GA	1280	1430	1400	1190
LA	220	195	280	170
MS	630	620	710	530
NM	66	77	63	43
NC	375	430	510	360
SC	250	300	300	190
TN	345	360	410	280
TX	7000	7750	7050	6800
12 cotton states	11,305	12,434	12,155	10,761
USA	12,465	13,850	13,508	11,890

Table S5. Percentage of corn acres planted with Vip3Aa corn.

State or Region	2017	2018	2019	2020
AL	0.7	0.9	6.5	8.7
AZ	4.7	5.5	9.3	16.5
AR	8.8	11.7	10.3	13.2
FL	23.2	14.5	18.2	12.0
GA	7.7	5.8	7.4	6.0
LA	1.6	4.5	3.2	3.3
MS	1.4	4.4	2.8	4.9
NM	4.5	6.0	6.2	10.5
NC	7.0	6.5	19.3	15.7
SC	4.7	5.4	14.7	19.1
TN	1.3	2.6	7.7	11.3
TX	7.9	11.7	14.8	21.3
12 cotton states	5.8	7.8	11.3	14.3
USA	3.0	3.4	5.2	6.4

Table S6. Percentage of cotton acres planted with Vip3Aa cotton.

State or Region	2017	2018	2019	2020
AL	4.5	11.3	22.7	30.2
AZ	9.4	20.3	32.3	60.8
AR	2.2	12.2	14.4	29.8
FL	1.1	3.1	12.8	19.2
GA	1.4	10.3	23.1	40.7
LA	3.5	13.3	28.7	34.1
MS	2.5	9.3	19.4	30.5
NM	78.6	19.1	31.9	37.7
NC	1.5	32.5	53.4	76.9
SC	4.4	8.9	21.7	39.7
TN	6.5	17.7	25.4	33.9
TX	2.4	8.9	26.9	32.9
12 cotton states	3.1	10.6	26.1	35.2
USA	3.7	11.7	27.4	36.3

Table S7. Larvae of H. zea per ear of Vip3Aa corn relative to comparable non-Bt corn in four previous field studies where the efficacy of the Vip3Aa corn can be attributed primarily or entirely to Vip3Aa.

Reference	Field site(s)	Year(s)	Larvae of *H. zea* per ear			Efficacy (%)[a]	Other Bt toxins in the Vip3Aa corn
			Vip3Aa corn	Non-Bt corn	Vip3Aa/ Non-Bt		
[19], Table 1	Snook, TX	2018	0.79	0.93	0.85	15.1	Cry1Ab, Cry1Fa[b]
[33], Table 1	Beltsville, MD	2007	0.055[c]	1.74	0.03	96.8	None
[33], Table 1	Beltsville, MD	2009	0.04	1.23	0.03	96.7	None
[17], Table 5	LA[d]	2018	0.06	0.935[e]	0.06	93.6	Cry1Ab, Cry1Fa[b]
[17], Table 5	LA[d]	2018	0.01	0.935[e]	0.01	98.9	Cry1A.105, Cry2Ab[b]
[21], p. 963	Winnsboro, LA	2018-19	0.0008[f]	0.77[g]	0.001	99.9	Cry1Ab[h]

a Efficacy = 100% X (1 – [larvae per Vip3Aa ear/Non-Bt ear])

b Corn containing these toxins and not Vip3Aa was not effective

c Mean for male and female plants as source of the Vip3Aa gene

d Data pooled for two sites, one in Franklin Parish, the other in Tensas Parish

e Mean for two non-Bt hybrids

f One third instar larvae seen in 1200 ears

g Based on pupae in pure non-Bt (Table 1), which could underestimate number of larvae and thus overestimate Vip3Aa/Non-Bt

h *H. zea* "highly resistant to Cry1Ab" in diet bioassays

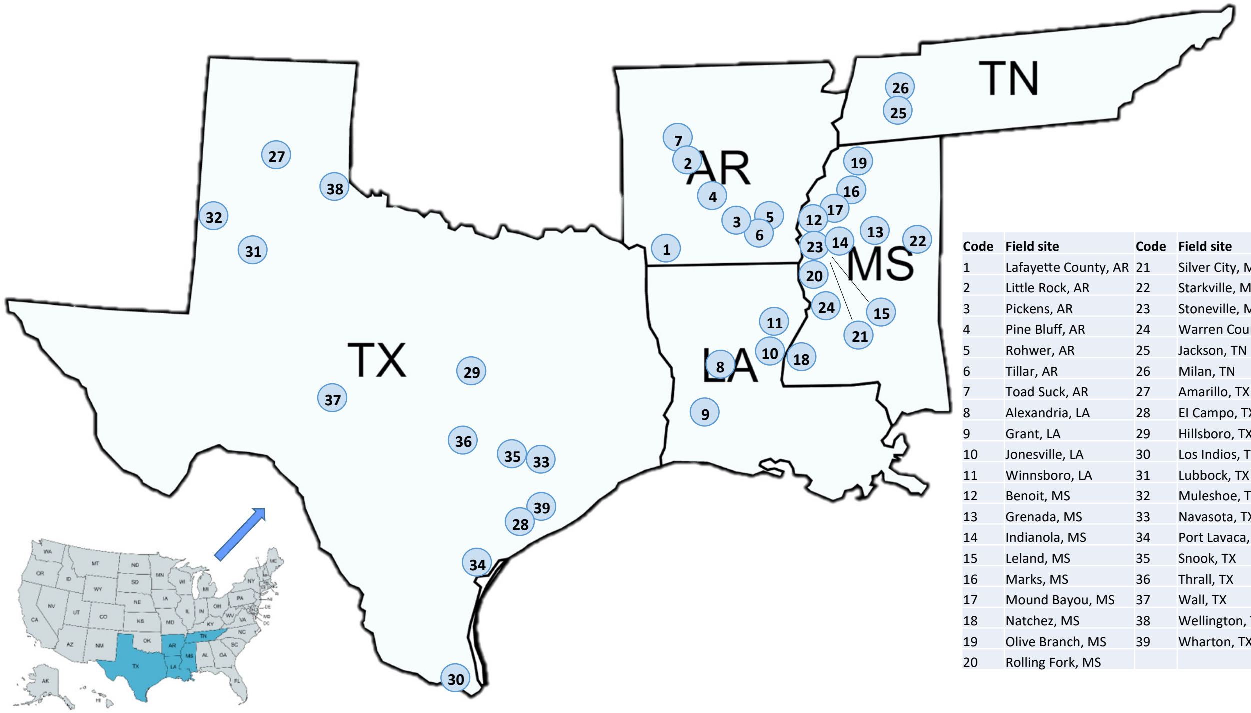

Code	Field site	Code	Field site
1	Lafayette County, AR	21	Silver City, MS
2	Little Rock, AR	22	Starkville, MS
3	Pickens, AR	23	Stoneville, MS
4	Pine Bluff, AR	24	Warren County, MS
5	Rohwer, AR	25	Jackson, TN
6	Tillar, AR	26	Milan, TN
7	Toad Suck, AR	27	Amarillo, TX
8	Alexandria, LA	28	El Campo, TX
9	Grant, LA	29	Hillsboro, TX
10	Jonesville, LA	30	Los Indios, TX
11	Winnsboro, LA	31	Lubbock, TX
12	Benoit, MS	32	Muleshoe, TX
13	Grenada, MS	33	Navasota, TX
14	Indianola, MS	34	Port Lavaca, TX
15	Leland, MS	35	Snook, TX
16	Marks, MS	36	Thrall, TX
17	Mound Bayou, MS	37	Wall, TX
18	Natchez, MS	38	Wellington, TX
19	Olive Branch, MS	39	Wharton, TX
20	Rolling Fork, MS		

Figure S1. Map of field sites for monitoring resistance of *H. zea* to Vip3Aa.

References

52. Schnell, R., K. Horn, E. Blair, and S. Murray. 2020. 2020 Texas Corn Performance Variety Trials. Texas A&M AgriLife. SCS-2020-11. https://varietytesting.tamu.edu/files/corn/2020/2020-CORN-PUBLICATION.PDF
53. Saavoss, M., T. Capehart, W. McBride, and A. Effland. 2021. Trends in Production Practices and Costs of the U.S. Corn Sector, ERR-2 USDA, Economic Research Service. https://www.ers.usda.gov/webdocs/publications/101722/err-294.pdf?v=2087.7
54. Robinson, E. Calculator compares cotton seed costs. 2003. FarmProgress. https://www.farmprogress.com/calculator-compares-cotton-seed-costs
55. U.S. Environmental Protection Agency (EPA). 2010. Acres Planted per Day and Seeding Rates of Crops Grown in the United States.https://www.epa.gov/sites/default/files/2018-01/documents/seeding-rates-and-acres-planted-per-day-revised-final-030111.pdf
56. USDA National Agricultural Statistics Service (NASS). 2020. Crop Production. https://www.nass.usda.gov/Publications/Todays_Reports/reports/cropan20.pdf
57. USDA National Agricultural Statistics Service (NASS). 2021. Acreage. https://www.nass.usda.gov/Publications/Todays_Reports/reports/acrg0621.pdf
58. Yu, J., Hennessy, D.A., Wu, F. The impact of Bt corn on aflatoxin-related insurance claims in the United States. *Sci. Rep.* **2020**, 10, 10046.
59. USDA Agricultural Marketing Service. Cotton Varieties Planted 2020 Crop. https://www.ams.usda.gov/mnreports/cnavar.pdf
